# Maize Ethylene Response Factor *ZmERF061* Is Required for Resistance to *Exserohilum turcicum*

**DOI:** 10.3389/fpls.2021.630413

**Published:** 2021-03-09

**Authors:** Zhenyuan Zang, Zhen Wang, Fuxing Zhao, Wei Yang, Jiabin Ci, Xuejiao Ren, Liangyu Jiang, Weiguang Yang

**Affiliations:** ^1^College of Agriculture, Jilin Agricultural University, Changchun, China; ^2^Crop Science Post-doctoral Station, Jilin Agricultural University, Changchun, China

**Keywords:** maize, *ZmERF061*, *Exserohilum turcicum*, transcription factor, defense response

## Abstract

Plants have evolved a series of sophisticated defense mechanisms to help them from harm. Ethylene Response Factor (ERF) plays pivotal roles in plant immune reactions, however, its underlying mechanism in maize with a defensive function to *Exserohilum turcicum* (*E. turcicum*) remains poorly understood. Here, we isolated and characterized a novel ERF transcription factor, designated *ZmERF061*, from maize. Phylogenetic analysis revealed that ZmERF061 is a member of B3 group in the ERF family. qRT-PCR assays showed that the expression of *ZmERF061* is significantly induced by *E. turcicum* inoculation and hormone treatments with salicylic acid (SA) and methyl jasmonate (MeJA). ZmERF061 was proved to function as a nucleus-localized transcription activator and specifically bind to the GCC-box element. *zmerf061* mutant lines resulted in enhanced susceptibility to *E. turcicum* via decreasing the expression of *ZmPR10.1* and *ZmPR10.2* and the activity of antioxidant defense system. *zmerf061* mutant lines increased the expression of the SA signaling-related gene *ZmPR1a* and decreased the expression of the jasmonic acid (JA) signaling-related gene *ZmLox1* after infection with *E. turcicum*. In addition, ZmERF061 could interact with ZmMPK6-1. These results suggested that ZmERF061 plays an important role in response to *E. turcicum* and may be useful in genetic engineering breeding.

## Introduction

Northern corn leaf blight (NCLB) caused by *Exserohilum turcicum* (*E. turcicum*) is one of the most destructive fungal diseases of maize in the world (Leach et al., 1977; Galiano-Carneiro and Miedaner, 2017). Heavy infections of NCLB can result in yield losses of greater than 50% (Fajemisin and Hooker, 1974; Raymundo and Hooker, 1981; Perkins and Pedersen, 1987; Ding et al., 2015). Additionally, NCLB may cause a reduction of feeding value and increase the incidence of maize to stalk rot (Hooker et al., 1965; Fajemisin and Hooker, 1974). Host plant resistance is the most common strategy used to control NCLB through the deployment of qualitative and quantitative resistance. Several qualitative *Ht* genes such as *Ht1*, *Ht2*, *Ht3*, *Htn1*, *HtM*, *HtP*, *HtNB*, *ht4*, and *rt* have already been identified and mapped in maize (Ullstrup, 1963; Gevers, 1975; Hooker, 1977, 1981; Bentolila et al., 1991; Robbins and Warren, 1993; Simcox and Bennetzen, 1993; Carson, 1995; Ogliari et al., 2005; Hurni et al., 2015; Galiano-Carneiro and Miedaner, 2017). However, these *Ht* genes can quickly get ineffective and result in significant yield losses due to the emergence of new, virulent races (Welz and Geiger, 2000; McDonald and Linde, 2002). Quantitative resistance is considered to provide more durable disease and more useful in breeding process than qualitative resistance (St Clair, 2010). Therefore, it is vital to identify the important genes conferring quantitative resistance and elucidate their molecular mechanism for improving disease resistance of maize cultivars against *E. turcicum*.

Plants have evolved a series of sophisticated defense mechanisms to cope with the invading pathogens during their life span (Jones and Dangl, 2006; Dangl et al., 2013; Fu and Dong, 2013; Bigeard et al., 2015; Birkenbihl et al., 2017). In plant immunity system, there are two layers of immune responses, called pathogen/microbe-associated molecular pattern (PAMP or MAMP)-triggered immunity (PTI) and effector-triggered immunity (ETI) (Cui et al., 2015; Couto and Zipfel, 2016; Zipfel and Oldroyd, 2017). During PTI and ETI responses, plants trigger a variety of immune reactions including the accumulation of reactive oxygen species (ROS), the activation of mitogen-activated protein kinase (MAPK) signaling pathway, and the expression of pathogenesis-related (PR) genes (Tsuda and Katagiri, 2010; Pieterse et al., 2012; Meng and Zhang, 2013; Baxter et al., 2014). In addition, ETI is often associated with programmed cell death (PCD), also called the hypersensitive response (HR), which occurs at the site of infection and prevents further invasion by the pathogen (Cui et al., 2015).

Transcription factors (TFs) play pivotal roles in plant immune reactions (Singh et al., 2002; Buscaill and Rivas, 2014; Huang et al., 2019). In recent studies, many TFs have been identified according to their conserved structural domain, such as APETALA2/Ethylene Response Factor (AP2/ERF), WRKY, NAC, and bZIP families (Eulgem and Somssich, 2007; Alves et al., 2013; Nuruzzaman et al., 2013; Huang et al., 2016). The AP2/ERF superfamily is a large plant-specific TF family and is defined by the conserved AP2/ERF domain that consists of 58 or 59 amino acids (Ohme-Takagi and Shinshi, 1995). The AP2/ERF superfamily is divided into the ERF family, AP2 family, and RAV family, based on the numbers and characteristics of the AP2/ERF domain (Licausi et al., 2013). The ERF family is further divided into two major subfamilies, DREBs and ERFs (Sakuma et al., 2002). ERF genes have been identified in many species, including *Arabidopsis* (Nakano et al., 2006), maize (Hao et al., 2020), rice (Nakano et al., 2006), wheat (Zhang et al., 2020), tomato (Yang et al., 2020), pepper (Jin et al., 2018), and soybean (Zhao et al., 2017). It has been well established that the ERF genes can specifically bind to the GCC-box element (AGCCGCC), which is present in the promoters of downstream defense-related genes (Ohme-Takagi and Shinshi, 1995; Fujimoto et al., 2000; Pré et al., 2008; Van der Does et al., 2013).

ERF genes as transcription activators or repressors are involved in modulating disease resistance reactions (Lu et al., 2013; Tian et al., 2015; Li et al., 2018). ERF transcription activators have been shown to positively regulate plant immune response against pathogens. For instance, overexpression of the transcription activators, *AtERF1*, Octadecanoid-Responsive *Arabidopsis* 59 (*ORA59*), *AtERF5*, *AtERF6*, *AtERF15*, and *AtERF96* in *Arabidopsis* resulted in a significantly enhanced resistance against *Botrytis cinerea* (*B. cinerea*) through activating the expression of defense related genes, including *PLANT DEFENSIN 1.2* (*PDF1.2*) (Berrocal-Lobo et al., 2002; Lorenzo et al., 2003; Pré et al., 2008; Moffat et al., 2012; Catinot et al., 2015; Zhang et al., 2015). ZmERF105 is a transcription activator, and overexpression of *ZmERF105* in maize enhanced resistance to *E. turcicum*, while the mutant of *zmerf105* led to decreased resistance (Zang et al., 2020). Overexpression of *TaPIE1*, a transcription activator, exhibited significantly increased resistance to *Rhizoctonia cerealis*, while *TaPIE1*-underexpressing wheat exhibited the opposite trend (Zhu et al., 2014). In tomato, SlERF.A1, SlERF.B4, or SlERF.C3 functions as a transcription activator and has been found to positively regulate the plant resistance against *B. cinerea* (Ouyang et al., 2016). In contrast, several ERF transcription repressors that contain an ERF-associated Amphiphilic Repression (EAR) motif in their C-terminal regions negatively regulated the plant resistance to pathogens (Ohta et al., 2000). The transcription repressors *AtERF4* and *AtERF*9 acted as negative regulators of resistance to *Fusarium oxysporum* and *B. cinerea*, respectively (McGrath et al., 2005; Maruyama et al., 2013).

ERF genes can coordinately integrate the salicylic acid (SA) and jasmonic acid (JA)/ethylene (ET) signaling pathways or antagonize them, to finely modulate the defense response to pathogens (Berrocal-Lobo and Molina, 2004; Zarei et al., 2011; Zhang et al., 2015, 2016; Wang et al., 2018). *AtERF1*, *AtERF96*, or *ORA59* has been shown to positively regulate the *Arabidopsis* defense against *B. cinerea* through the JA/ET signaling pathway and negatively modulate immunity against *Pseudomonas syringae* pv. *tomato* (*Pst*) DC3000 through the SA signaling pathway (Berrocal-Lobo et al., 2002; Pré et al., 2008; Catinot et al., 2015). It was also found that *AtERF15* is involved in resistance to *Pst* DC3000 and *B. cinerea* via the SA and JA/ET signaling pathways (Zhang et al., 2015). *VqERF112*, *VqERF114*, and *VqERF072* acted as positive regulators of plant resistance against *Pst* DC3000 and *B. cinerea* through integrating the SA and JA/ET signaling pathways (Wang L. et al., 2020). *AtERF11* positively regulated *Arabidopsis* resistance to *Pst* DC3000 by directly activating the transcription of *AtBT4*, which depends on the SA and ET signaling pathways (Zheng et al., 2019).

Recently, several ERF genes have shown to regulate the expression of their target genes through interaction with other proteins (Huang et al., 2016). ORA59 physically interacted with RELATED TO AP2.3 (RAP2.3) to increase the plant resistance against *Pectobacterium carotovorum* (Kim et al., 2018). GmERF5 and GmERF113 interacted with a BASIC HELIX-LOOP-HELIX TF (GmbHLH) to improve the soybean resistance against *Phytophthora sojae* (*P. sojae*) (Dong et al., 2015; Zhao et al., 2017). AtERF6 could interact with AtMPK6 and directly be phosphorylated by AtMPK6. The phosphorylation of AtERF6 increased its protein stability and thus constitutively activated defense genes (Meng et al., 2013; Wang et al., 2013).

A large number of ERF genes have been shown to regulate plant resistance against pathogens in many species, however, its underlying mechanism in maize with a defensive function to *E. turcicum* remains poorly understood. Previously, we identified a maize ERF gene whose expression was specifically induced by *E. turcicum* inoculation. Therefore, we isolated and characterized *ZmERF061* from maize B73. ZmERF061 was proved to function as a nucleus-localized transcription activator and specifically bind to the GCC-box element. *zmerf061* mutant lines resulted in decreased resistance to *E. turcicum*. In addition, ZmERF061 could interact with ZmMPK6-1. These results suggested that ZmERF061 plays an important role in response to *E. turcicum* and may be useful in genetic engineering breeding.

## Materials and Methods

### Plant Materials and Treatments

*Exserohilum turcicum* (mixed races), the seeds of the maize inbred lines Mo17 (resistant to *E. turcicum*), Huobai (resistant to *E. turcicum*), and B73 were obtained from Maize Breeding Team in Jilin Agricultural University, Changchun, China. The seedlings were grown in a glasshouse at 25°C under long-day (16 h light/8 h dark) and 70% relative humidity conditions. For hormone treatments, the seedlings of maize inbred line B73 were sprayed with 0.1 mM of methyl jasmonate (MeJA) and 0.5 mM of SA at the three-leaf stage. For *E. turcicum* inoculation, the seedlings of maize inbred line Mo17 and Huobai were inoculated with three drops of conidial suspensions at the six-leaf stage according to the method of Zang et al. (2020). The conidial suspensions were adjusted to 1 × 10^5^ conidia ml^–1^. The leaves were sampled at 0, 2, 5, 10, and 24 h after hormone treatments and were collected at 0, 10, 24, and 72 h after *E. turcicum* inoculation, respectively. The leaves were frozen in liquid nitrogen and stored at -80°C for the subsequent quantitative real-time polymerase chain reaction (qRT-PCR) analysis. The special primers used for assays are listed in Supplementary Table S1.

### qRT-PCR Analysis

Total RNA was extracted from maize leaves using TRIzol reagent (Invitrogen, China) according to the manufacturer’s instruction. Total RNA (1 μg) was used to reverse transcribe into complementary DNA (cDNA) with ReverTra Ace^®^, qPCR RT Kit (TOYOBO, Japan) following the manufacture’s instruction. qRT-PCR was performed using SYBR Mixture system (TOYOBO, Japan) on a QuantStudio 3 instrument (Thermo, United States). A maize Actin gene, *ZmTub* (GRMZM2G066191), was used as an internal control to normalize the data. The relative expression levels of genes were analyzed using the 2^–Δ^ ^Δ^ ^*CT*^ method. The experimental data were determined using three independent biological repeats, and the significance analysis was performed using Student’s *t*-test (^∗^*P* < 0.05, ^∗∗^*P* < 0.01). Bars indicate standard error of the mean.

### Cloning and Bioinformatics Analysis of *ZmERF061*

The full-length coding sequence of *ZmERF061* was isolated from the leaves of B73 by reverse transcription PCR (RT-PCR). The PCR product was cloned into the pMD18-T vector (TaKaRa, China), and the sequence was verified by sequencing (Sangon, China). ERF sequences from different species were downloaded from the NCBI database^1^, and the phylogenetic tree was built with MEGA 5.0 software using the neighbor joining (NJ) method. The amino acid sequence alignment was performed by DNAMAN software. The nucleic acid sequence and protein sequence of *ZmERF061* were analyzed using ExPASy^2^ database.

### Yeast Two-Hybrid Assay

The full-length coding sequence of *ZmERF061* was inserted into pGBKT7 vector to generate the bait plasmid (pGBKT7-ZmERF061). The coding sequence of *ZmMPK6-1* was cloned into pGADT7 vector to generate prey plasmid (pGADT7-ZmMPK6-1). The prey and bait plasmids were co-transformed into the yeast strain Y_2_H according to the manufacturer’s instructions (Clontech, United States). After selection on SD/-Trp/-Leu medium for 3 days at 30°C, the transformants were grown on SD/-Trp/-Leu/-His/-Ade medium containing X-α-Gal (20 μg ml^–1^). Yeast cells carrying the pGBKT7-p53 and pGADT7-SV40 plasmids were used as positive controls, and yeast cells harboring the pGBKT7-Lam and pGADT7-SV40 plasmids were used as negative controls.

### Subcellular Localization and Bimolecular Fluorescence Complementation Assays

For subcellular localization of ZmERF061, fusion expression vector ZmERF061–green fluorescent protein (GFP) was constructed by inserting full-length coding sequence of *ZmERF061* into the pCAMBIA1300 vector. For bimolecular fluorescence complementation (BiFC) assays, the full-length coding sequences of ZmERF061 and ZmMPK6-1 were fused into pUC-SPYCE and pUC-SPYNE vectors, respectively. The plasmids were transiently expressed in *Nicotiana benthamiana* (*N. benthamiana*) leaves by *Agrobacterium*-mediated method (Liu et al., 2010). The fluorescence signal in cells was photographed by a laser confocal microscope (Leica TCS SP2, Germany).

### Yeast One-Hybrid Assay

Yeast one-hybrid assays were used to examine the binding of ZmERF061 to a GCC-box element and were performed according to the Matchmaker Gold Yeat One-Hybrid Library Screening System (Clontech, United States). The full-length coding sequence of *ZmERF061* was cloned into the pGADT7 vector containing a GAL4 transcription activation domain, to generate the prey plasmid (pGADT7-ZmERF061). The synthesized DNA fragments harboring four tandem copies of the GCC-box (ATCCATAAGAGCCGCCACTAAAATAAGACCGATCAA) and mGCC (ATCCATAAGATCCTCCACTAAAATAAGACCGATC AA) were cloned into the pAbAi vector as bait plasmids (pAbAi-4 × GCC and pAbAi-4 × mGCC), respectively. The pGADT7-ZmERF061 plasmid was co-transformed with pAbAi-4 × GCC and pAbAi-4 × mGCC plasmids, into Y_1_H Gold yeast strain, respectively. The co-transformation yeasts were determined on SD/-Leu/-Ura medium supplemented with 200 ng ml^–1^ of AbA or 300 ng ml^–1^ of AbA and cultured at 30°C for 3 days. Positive (pGAD-rec-53 + pAbAi-p53) and negative (pGADT7 + pAbAi) controls were processed in the same manner.

### Luciferase Activity Assay

The full-length coding sequence of *ZmERF061* was cloned into the pGreenII 62-SK vector as effector, and four tandem copies of the GCC-box (ATCCATAAGAGCCGCCACTAAAATAAGACCGATCAA) were ligated into the pGREENII0800-LUC vector as reporter (4 × GCC-LUC). The effector and reporter plasmids were, respectively transferred into *Agrobacterium tumefaciens* GV3101 and co-transformed into *N. benthamiana* leaves by *Agrobacterium*-mediated method (Liu et al., 2010). The LUC activity was determined using commercial dual-LUC reaction reagents (Promega, United States) according to the previous report (Ma et al., 2018). Empty pGreenII 62-SK vector co-transformed with 4 × GCC-LUC was used as the negative control.

### Pathogen Response Assays of *zmerf061* Mutant Lines

Loss-of-function *zmerf061* mutant lines (*zmerf061* UFMu mutant, mu1014012) were obtained from the Maize Genetics Cooperation Stock Center. The homozygous mutant lines were obtained from self-fertilizing and identified by PCR. Then, two homozygous T_4_ *zmerf061* mutant lines, named *zmerf061-1* and *zmerf061-2*, were confirmed by qRT-PCR and used for further analyses. Artificial inoculation procedures were performed according to the methods described by Zang et al. (2020). The living ear leaves of *zmerf061* mutant lines were infected with *E. turcicum* agar disks, and the detached leaves from inoculated plants were pictured at 5 days post-inoculation (dpi) with a Nikon D7000 camera for disease assays. The relative lesion area was evaluated using the Photoshop CS3 software according to Cui et al. (2009).

### Detection of Enzyme Activities

For the enzyme activity assays, the fresh leaves (about 0.1 g) of W22 wild-type (WT) plants and *zmerf061* mutant lines were harvested 24 h after inoculation with *E. turcicum* conidial suspension, and the plants that were treated with water served as control. The superoxide dismutase (SOD) and peroxidase (POD) activities were measured following the methods that described by Li et al. (2015).

## Results

### Cloning and Characterization of *ZmERF061*

**FIGURE 1:**
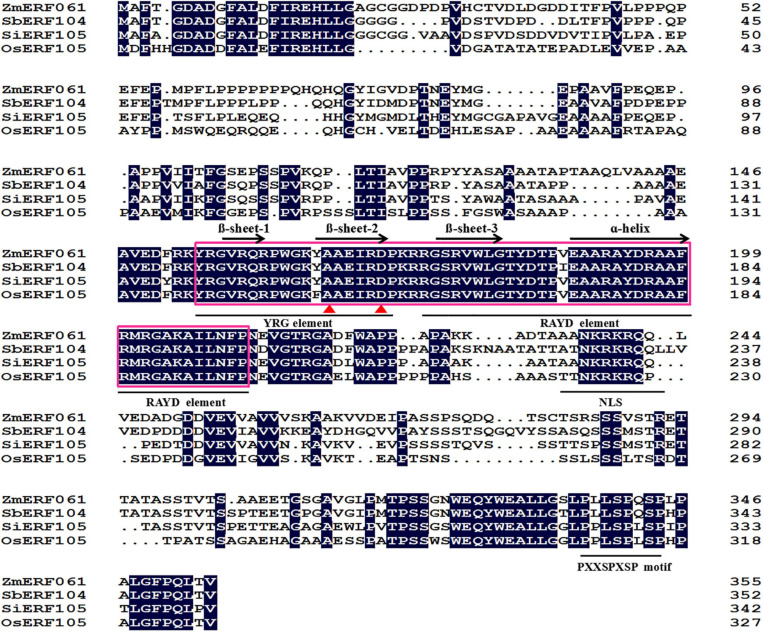
Alignment of the ZmERF061 with other Ethylene Response Factor (ERF) proteins. The sequence alignment was performed using DNAMAN software. The AP2/ERF domain is indicated by a pink box. The one α-helix and three β-sheets are marked above the corresponding sequences. The YRG and RAYD elements are indicated with a black horizontal solid line. The conserved alanine and aspartic acid residues are marked by red triangles. NLS and PXXSPXSP motifs are marked with a black horizontal solid line. OsERF105 (XP015635116) is derived from *Oryza sativa*, SbERF104 (XP021319691) is derived from *Sorghum bicolor*, and SiERF105 (XP004976417) is derived from *Setaria italica*.

**FIGURE 2:**
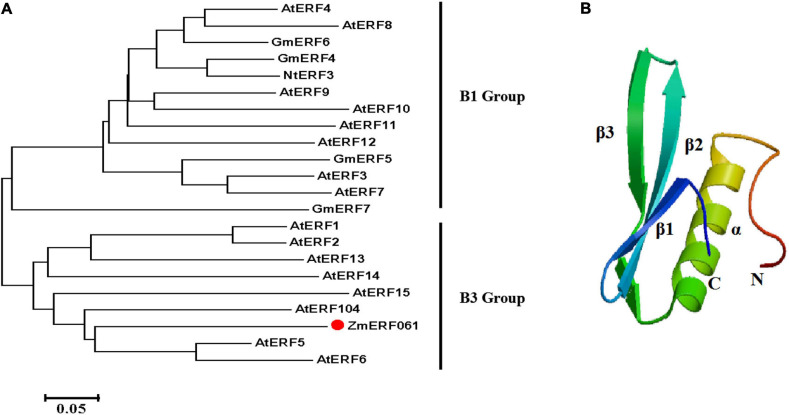
Phylogenetic analysis and three-dimensional structure of ZmERF061. **(A)** The phylogenetic tree was constructed with the MEGA 5.0 software using the neighbor joining method. ZmERF061 is indicated by the red dot. The accession numbers are as follows: AtERF1 (NP188965), AtERF2 (NP199533), AtERF3 (NP175479), AtERF4 (NP188139), AtERF5 (NP568679), AtERF6 (NP567529), AtERF7 (NP188666), AtERF8 (NP175725), AtERF9 (NP199234), AtERF10 (NP171876), AtERF11 (NP174159), AtERF12 (NP174158), AtERF13 (NP182011), AtERF14 (NP171932), AtERF15 (NP9850162), AtERF104 (NP_200968), GmERF4 (ACE76905), GmERF5 (AEX25891), GmERF6 (AEQ55267), GmERF7 (AEQ55266), and NtERF3 (BAJ72664). **(B)** Predicted three-dimensional structure of ZmERF061.

*ZmERF061* (GenBank Accession no. XM008670839), the ERF gene, was isolated from total RNA of maize by RT-PCR. Sequence analysis revealed that *ZmERF061* contains a 1,071-bp open reading frame (ORF) encoding a polypeptide of 356 amino acids (aa) with predicted molecular mass of 37.783 kDa (pI 4.84). The results from searching the database^3^ indicated that *ZmERF061* is located on chromosome 2 and does not have signal peptide. ZmERF061 contains a typical AP2/ERF domain, with conserved alanine (A) and aspartic acid (D) in it, suggesting that it belongs to the ERF family (Sakuma et al., 2002). The AP2/ERF domain contains conserved YRG and RAYD elements, which have been shown to play a vital role in GCC-box binding activity and protein interaction, respectively (Mazarel et al., 2002). ZmERF061 contains a conserved PXXSPXSP (X represents any amino acid) motif in the C-terminal region, which is believed to act as MPK phosphorylation sites (Meng et al., 2013). Additionally, ZmERF061 also possessed a nuclear targeting signal (NLS) sequence “AANKRKRQQL” (Figure 1). Blast search in NCBI revealed that ZmERF061 shares 67.75, 65.38, and 52.75% identity to SbERF104 (Protein ID: XP021319691), SiERF105 (Protein ID: XP004976417), and OsERF105 (Protein ID: XP015635116), respectively (Figure 2A). The phylogenetic tree analysis indicated that ZmERF061 belongs to B3 group (Sakuma et al., 2002). The prediction of the three-dimensional structure based on SWISS-MODEL database revealed that the ZmERF061 has a long C-terminal α-helix (α) surrounded by a three-stranded anti-parallel β-sheet (from β1 to β3) (Figure 2B).

### Expressions of *ZmERF061* Responds to Pathogen Infection and Hormone Induction

**FIGURE 3:**
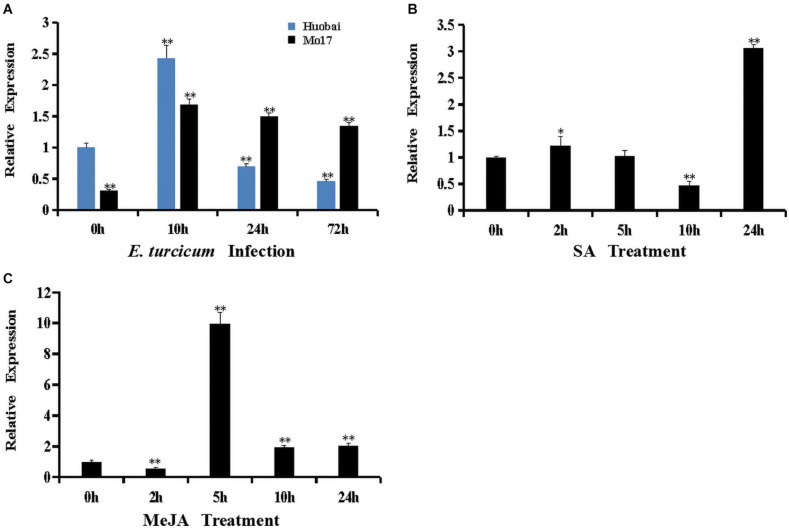
Expression of *ZmERF061* was induced by *Exserohilum turcicum* inoculation and by hormone treatments with salicylic acid (SA) and methyl jasmonate (MeJA). **(A)** Expression of *ZmERF061* in Mo17 and Huobai after inoculation with *E. turcicum*. The samples were collected at 0, 10, 24, and 72 h after *E. turcicum* infection. Relative expression levels were compared with Huobai at 0 h. **(B)** Expression of *ZmERF061* in B73 after treatment with 0.5 mM of SA. **(C)** Expression of *ZmERF061* in B73 after treatment with 0.1 mM of MeJA. The samples were collected at 0, 2, 5, 10, and 24 h after the initiation of treatments. Transcript levels were normalized to *ZmTub* (GRMZM2G066191). The relative expression levels of genes were analyzed using the 2^– Δ^ ^Δ^ ^*CT*^ method. The experimental data were determined using three independent biological replicates, and the significance analysis was performed using Student’s *t*-test (*P < 0.05, **P < 0.01). Bars indicate standard error of the mean.

To characterize the potential role of *ZmERF061* in plant defense reaction, the expression profiles of *ZmERF061* in both resistant maize inbred line Mo17 and susceptible maize inbred line Huobai following inoculation with *E. turcicum* were analyzed by qRT-PCR. The expression level of *ZmERF061* in maize inbred Huobai was increased at 10 h but rapidly decreased at 24 h (0.69-fold) and 72 h (0.46-fold) compared with Huobai, however a significant upregulation of *ZmERF061* expression is detected in the leaves from 10 to 72 h after *E. turcicum* in maize inbred line Mo17 (Figure 3A). These results indicated that *ZmERF061* may play an important role in maize defense response to *E. turcicum*.

ERF genes are involved in a variety of defense signaling hormones, such as SA and JA. In our qRT-PCR assay, the transcript levels of *ZmERF061* were analyzed in maize inbred line B73 after the application of SA and MeJA treatments. The expression of *ZmERF061* was decreased at 2 h, then rapidly increased from 5 to 24 h, and peaked at 5 h by 9.96-fold after the application of MeJA treatment. In SA-treated plants, the expression level of *ZmERF061* was lower at 10 h but peaked 3.06-fold higher at 24 h than that in the control. These results indicated that *ZmERF061* responds to *E. turcicum* inoculation and is involved in the JA and SA signaling pathways (Figures 3B,C).

### Subcellular Localization of ZmERF061

**FIGURE 4:**
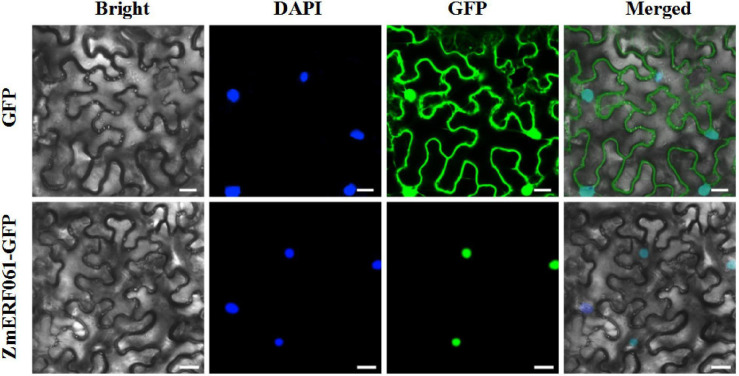
Subcellular localization of the ZmERF061 in *Nicotiana benthamiana*. Transient expression of the green fluorescent protein (GFP) (upper) and ZmERF061–GFP (bottom) in *N. benthamiana*. Fluorescence were observed using a laser confocal microscope. Bars = 25 μm.

To elucidate the biological role of ZmERF061, the subcellular localization was analyzed *in planta*. The coding sequence of *ZmERF061* was fused to GFP and was transiently expressed in leaf epidermal cells of *N. benthamiana*. The ZmERF061–GFP fusion protein was solely localized in nucleus, while the control GFP protein was located in both nucleus and cytoplasm. This result indicated that ZmERF061 is a nucleus-localized protein (Figure 4).

### ZmERF061 Binds to GCC-Box Element and Functions as a Transcriptional Activator

**FIGURE 5:**
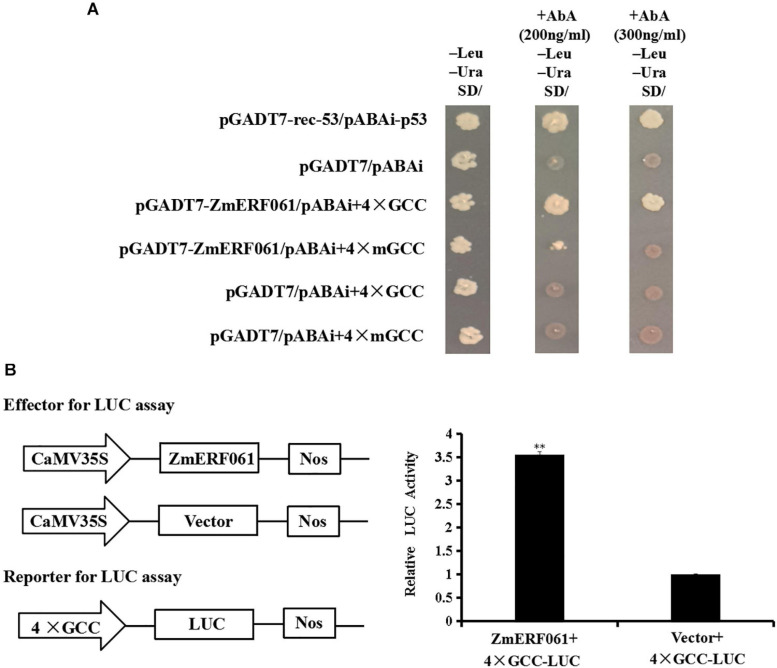
The binding activity of ZmERF061 to GCC-box element and transactivation activity. **(A)** The binding characteristics of ZmERF061 to GCC-box element. The full-length coding sequence of *ZmERF061* was fused with the GAL4 transcription activation domain of vector pGADT7 to generate the prey plasmid (pGADT7-ZmERF061). Four tandem copies of the GCC-box and mGCC-box mutants were cloned into the pAbAi vector and used as bait. The yeast transformants were incubated for 3 days at 30°C on SD/-Leu/-Ura medium with 200 ng ml^– 1^ of AbA or 300 ng ml^– 1^ of AbA. Positive (pGAD-rec-53 + pAbAi-p53) and negative (pGADT7 + pAbAi) controls were processed in the same manner. **(B)** Transcription activity of ZmERF061. The full-length coding sequence of *ZmERF061* was cloned into the pGreenII 62-SK vector as effector, and four tandem copies of the GCC-box were ligated into the pGREENII0800-LUC vector as reporter. Relative luciferase activity detected by transient co-transformation with reporter and effector into *N. benthamiana*. The experiment was performed using three independent biological replicates and analyzed using Student’s *t*-tests (**P < 0.01). Bars indicate standard error of the mean.

Previous studies have demonstrated that some ERF genes can bind to the GCC-box element (Guo et al., 2004; Franco-Zorrilla et al., 2014; Sun et al., 2016). A yeast one-hybrid assay was performed to investigate the binding characteristics of ZmERF061 to the GCC-box element. The yeast cells transfected with pGADT7-ZmERF061 and pAbAi-4 × GCC could grow on SD/-Leu/-Ura medium containing 200 ng ml^–1^ of AbA or 300 ng ml^–1^ of AbA. By contrast, yeast cells harboring the mutant bait cannot grow normally. These data suggested that ZmERF061 specifically binds to the GCC-box element (Figure 5A).

To determine whether ZmERF061 plays a role in transcription activation or inhibition, we performed a transient LUC assay. As shown in Figure 5B, the relative LUC activity of tobacco leaves transfected with the ZmERF061 effector and 4 × GCC-LUC reporter was approximately 3.55-fold higher than that of the control, indicating that ZmERF061 can activate the reporter gene transcription. These results demonstrated that ZmERF061 is able to bind to the GCC-box element and functions as a transcription activator.

### *zmerf061* Mutant Lines Decreased the Resistance to *Exserohilum turcicum*

**FIGURE 6:**
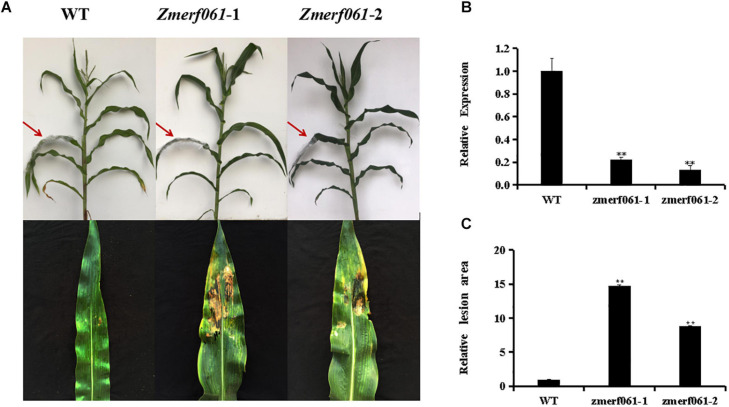
*zmerf061* mutant lines decreased the resistance to *Exserohilum turcicum*. **(A)** Disease symptom on detached leaves at 5 days post inoculation (dpi). **(B**) Relative expression analysis of *ZmERF061* in *zmerf061* mutant lines. **(C)** The relative lesion areas of *ZmERF061* mutant lines and wild-type (WT) leaves at 5 dpi. The experiment was performed using three independent biological replicates and analyzed using Student’s *t*-tests (**P < 0.01). Bars indicate standard error of the mean.

To explore the role of *ZmERF061* in mediating the maize resistance to *E. turcicum*, loss-of-function *zmerf061* UFMu mutant lines were obtained from the Maize Genetics Cooperation Stock Center. Homozygous *zmerf061* mutant lines were obtained from self-fertilizing and were characterized by PCR. Two homozygous T_4_ *zmerf061* mutant lines, named *zmerf061-1* and *zmerf061-2*, were confirmed by qRT-PCR. qRT-PCR analysis revealed that the expression levels of *ZmERF061* have about 0.22-fold and 0.14-fold declines in *zmerf061-1* and *zmerf061-2* mutant lines, respectively (Figure 6B). WT plants and two independent T_4_ *zmerf061* mutant lines were inoculated with *E. turcicum* to examine whether *ZmERF061* is involved in pathogen resistance. The detached leaves from inoculated plants were pictured with a Nikon D7000 camera for disease assays. At 5 dpi, *E. turcicum*-caused lesions were significantly smaller on the leaves of the WT plants compared with the *zmerf061* mutant lines, indicating that *zmerf061* mutant lines decreased resistance to *E. turcicum* (Figures 6A,C). These results demonstrate that *ZmERF061* positively regulates the maize resistance against *E. turcicum*.

### *ZmERF061* Mutant Lines Attenuated *Exserohilum turcicum*-Induced Defense Response

**FIGURE 7:**
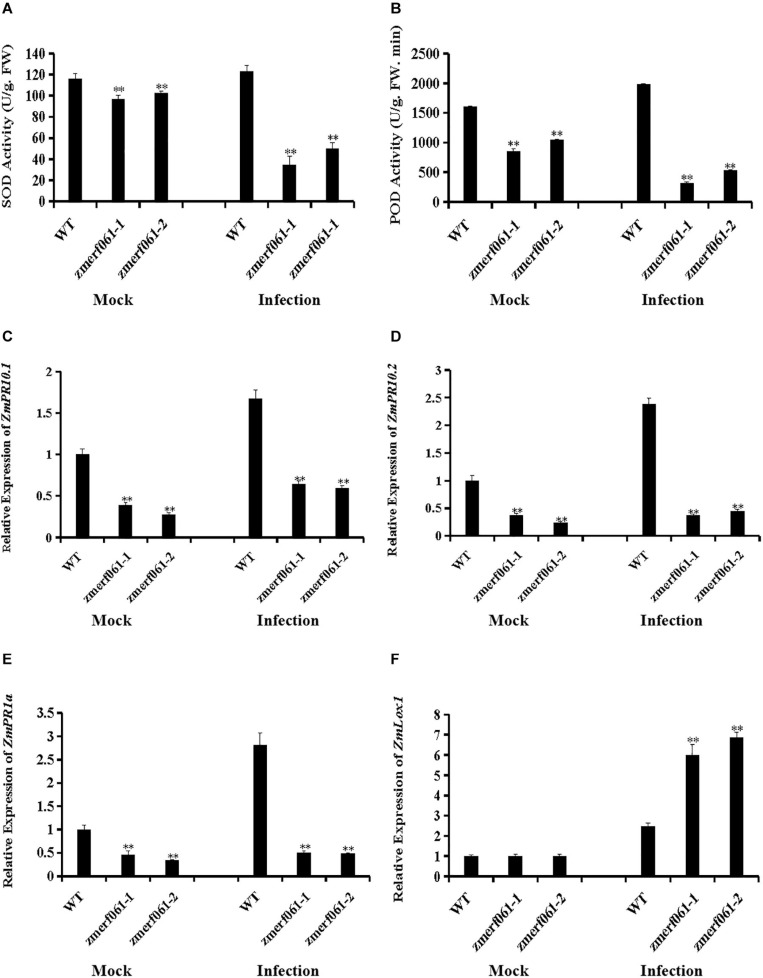
Altered antioxidant enzyme accumulation and defense gene expression in *zmerf061* mutant lines after infection with *Exserohilum turcicum*. **(A,B)** The activities of superoxide dismutase (SOD) and peroxidase (POD) in *zmerf061* mutant lines and wild-type (WT) plants at 24 h after *E. turcicum* inoculation. **(C,D)** Expression analysis of *ZmPR10.1* and *ZmPR10.2* in *zmerf061* mutant lines and WT plants at 24 h after *E. turcicum* inoculation. **(E,F)** Expression analysis of salicylic acid (SA)-responsive gene *ZmPR1a* and jasmonic acid (JA)-responsive gene *ZmLox1* in *zmerf061* mutant lines and WT plants at 24 h after *E. turcicum* inoculation. The mock-treated WT sample was set to unity. Transcript levels of *ZmPR10.1*, *ZmPR10.2*, *ZmPR1a*, and *ZmLox1* were normalized to *ZmTub* (GRMZM2G066191). The relative expression levels of genes were analyzed using the 2^– Δ^ ^Δ^ ^*CT*^ method. The experiment was performed using three independent biological replicates and analyzed using Student’s *t*-tests (**P < 0.01). Bars indicate standard error of the mean.

To further investigate the physiological changes in *zmerf061* mutant lines after infection with *E. turcicum*, we analyzed the activities of two important antioxidant enzymes, including SOD and POD. Under both the mock treatment and at 24 h after infection with *E. turcicum*, both SOD and POD activities were significantly decreased in *zmerf061* mutant lines compared with WT plants (Figures 7A,B). These results indicate that *ZmERF061* improves maize resistance against *E. turcicum* through affecting SOD and POD activities.

To examine whether the increased susceptibility to *E. turcicum* in *zmerf061* mutant lines was associated with transcription changes of defense-related genes, we measured the expression levels of two defense-related genes [i.e., *ZmPR10.1* (GRMZM2G112488) and *ZmPR10.2* (GRMZM2G112538)] in the WT plants and *zmerf061* mutant lines after *E. turcicum* inoculation. Under both the mock treatment and at 24 h inoculation with *E. turcicum*, the expression levels of *ZmPR10.1* and *ZmPR10.2* were significantly lower in *zmerf061* mutant lines than in the WT plants (Figures 7C,D). These results showed that *ZmERF061* is involved in maize resistance to *E. turcicum* through regulating the expression of defense-related genes.

To explore whether *ZmERF061* is required for SA-induced and JA-induced defense response, the expression levels of SA- and JA-responsive genes were analyzed by qRT-PCR after *E. turcicum* inoculation. Under both the mock treatment and at 24 h after infection with *E. turcicum*, the expression levels of SA-responsive gene, *ZmPR1a* (GRMZM2G465226), were significantly lower in *zmerf061* mutant lines than in the WT plants (Figure 7E). By contrast, the expression levels of the JA signaling-related gene, *ZmLox1* (GRMZM2G156861), were increased after *E. turcicum* inoculation in *zmerf061* mutant lines compared with the WT plants (Figure 7F). These results demonstrated that *ZmERF061* may regulate resistance against *E. turcicum* via the SA signaling pathway.

### *ZmERF061* Interacted With ZmMPK6-1

Recently, several B3 group ERF TFs have been shown to interact with MPK6 and involve in MAPK signaling cascade (Bethke et al., 2009; Wang et al., 2013). Amino acid sequence analysis has suggested that ZmERF061 contains putative MPK phosphorylation sites at its C terminus (Fujimoto et al., 2000). Thus, we anticipated that ZmERF061 might interact with ZmMPK6-1 (GRMZM2G002100), which shares high identity to AtMPK6 (At2g43790).

**FIGURE 8:**
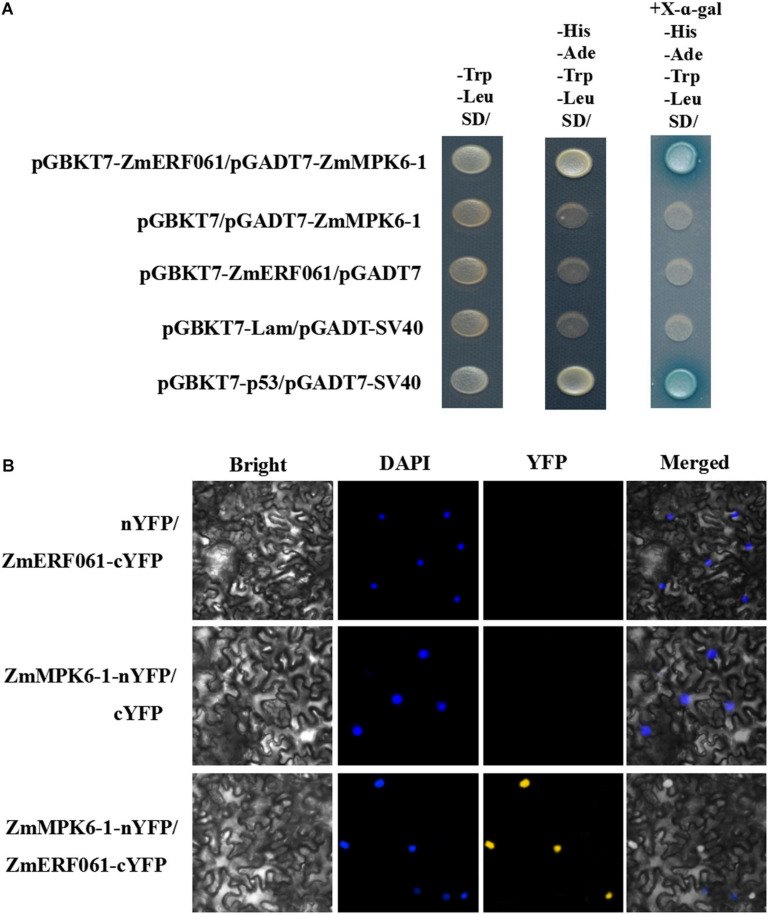
ZmERF061 physically interacts with ZmMPK6-1 in yeast cells and *Nicotiana benthamiana*. **(A)** The full-length ZmERF061 interacts with the ZmMPK6-1 in yeast cells. Transformed cells were spotted on the SD (-Leu/-Trp) medium, selective SD (-Leu/-Trp/-His/-Ade) medium, and selective SD (-Leu/-Trp/-His/-Ade) medium containing X-a-Gal (20 μg ml^–1^). Yeast Y_2_H gold cells carrying pGBKT7-p53 and pGADT7-SV40 served as a positive control, whereas co-expression of pGBKT7-Lam and pGADT7-SV40 was used as a negative control. **(B)** Bimolecular Fluorescence Complementation (BiFC) assay verifies the interaction between ZmERF061 and ZmMPK6-1 in *N. benthamiana*. ZmERF061-cYFP and ZmMPK6-1-nYFP were co-transfected into *N. benthamiana*. Fluorescence was observed using a laser confocal microscope. Bars = 25 μm.

To test this hypothesis, yeast two-hybrid assays were performed. In yeast two-hybrid assay, the yeast cells co-transformed with pGBKT7-ZmERF061 and pGADT7-ZmMPK6-1 developed well on SD (-Trp/-Leu/-His/-Ade) medium containing X-a-Gal (20 μg ml^–1^), indicating that ZmERF061 can interact with ZmMPK6-1 (Figure 8A).

To confirm the interaction between ZmERF061 and ZmMPK6-1, a BiFC assay was performed in leaf epidermal cells of *N. benthamiana*. As shown in Figure 8B, yellow fluorescence was displayed in leaf epidermal cells of *N. benthamiana* co-transformed with N-terminal yellow fluorescent protein (nYFP)-tagged ZmMPK6-1 and C-terminal YFP (cYFP)-tagged ZmERF061. These results indicated that ZmERF061 physically interacts with ZmMPK6-1.

## Discussion

ERF TFs play critical roles in response to pathogen infection in plants. In maize, a total of 76 predicted ERF genes have been identified (Hao et al., 2020). In the present study, a novel ERF gene, *ZmERF061*, was firstly isolated and functionally characterized in maize. Sequence analysis showed that ZmERF061 is characterized as a member of B3 group in the ERF family (Figure 2A). Nucleus-localized ZmERF061 plays a positive role in plant resistance to *E. turcicum* (Figures 4, 6).

Recent investigations demonstrated that a number of ERF TFs as positive regulators involved in plant defense response against pathogens (Liang et al., 2008; Son et al., 2012; Lu et al., 2013). Constitutive overexpression of *AtERF1*, *ORA59*, *AtERF5*, *AtERF6*, or *AtERF96* in *Arabidopsis* was shown to confer enhanced resistance to *B. cinerea* (Berrocal-Lobo et al., 2002; Lorenzo et al., 2003; Pré et al., 2008; Moffat et al., 2012; Catinot et al., 2015). *Arabidopsis* plants overexpressing *AtERF11* resulted in increased *Pst DC3000* resistance, and loss-of-function of *AtERF11* decreased *Arabidopsis* resistance to *Pst DC3000* (Zheng et al., 2019). Overexpression of *AtERF15*, *AcERF2*, *VqERF112*, *VqERF114*, or *VqERF072* in transgenic *Arabidopsis* showed improved resistance to *Pst DC3000* and *B. cinerea* (Zhang et al., 2015; Sun et al., 2018; Wang L. et al., 2020). Transgenic *Arabidopsis* overexpression of *MdERF11* led to enhanced resistance against *Botryosphaeria dothidea* (Wang J. H. et al., 2020). Silencing of *SlERF.A1*, *SlERF.A3*, *SlERF.B4*, or *SlERF.C3* in tomato exhibited decreased resistance against *B. cinerea* (Ouyang et al., 2016). *GmERF113* and *GmERF5* positively regulated the soybean resistance to *P. sojae* (Dong et al., 2015; Zhao et al., 2017). Similar to these results, the expression levels of *ZmERF061* were specifically induced by *E. turcicum* inoculation in maize inbred line Mo17 and Huobai (Figure 3A). *zmerf061* mutant lines resulted in enhanced susceptibility against *E. turcicum* (Figure 6). The expression levels of defense-related genes (Z*mPR10.1* and *ZmPR10.2*) were significantly compromised in *zmerf061* mutant lines, compared with WT plans in response to *E. turcicum* infection (Figures 7C,D). These results demonstrated that ZmERF061 positively modulates immunity against *E. turcicum* in maize. In contrast, some ERF TFs also negatively regulate the resistance to pathogens. For example, *OsERF922*-overexpressing plants decreased the resistance to *Magnaporthe oryzae*, while RNAi-mediated suppression *OsERF922* showed increased resistance (Liu et al., 2012). Knockout mutants of *AtERF9* showed enhanced resistance to *B. cinerea* (Maruyama et al., 2013). Overexpression of *AtERF014* decreased the resistance to *B. cinerea* (Zhang et al., 2016). *AtERF19* negatively regulated *Arabidopsis* resistances to *B. cinere*a and *Pst DC3000* (Huang et al., 2019).

Previous studies have shown that ERF genes function in plants’ immune response through modulating diverse hormone signaling molecules, such as SA, JA, and ET (Zarei et al., 2011; Zhang et al., 2016). Generally, ERF TFs regulate the *Arabidopsis* defense against necrotrophic pathogens through the JA/ET signaling pathway and negatively modulate immunity against (hemi)biotrophic pathogens through the SA signaling pathway (Berrocal-Lobo et al., 2002; Pré et al., 2008; Catinot et al., 2015). However, our present study demonstrated that relative expression levels of *ZmERF061* were significantly induced by SA and the expression levels of SA-responsive defense gene (*ZmPR1a*) were decreased in *zmerf061* mutant lines after infection with *E. turcicum*, indicating that *ZmERF061* may be involved in *E. turcicum* resistance via the SA signaling pathway (Figures 3B, 7E). This is different from the functions of *AtERF1*, *AtERF5*, *AtERF6*, *ORA59*, or *AtERF96*, which have been reported to play positive roles in disease resistance against *B. cinerea* by promoting the JA/ET signaling pathway (Berrocal-Lobo et al., 2002; Lorenzo et al., 2003; Pré et al., 2008; Moffat et al., 2012; Catinot et al., 2015). In addition, we demonstrated that the expression levels of JA-responsive defense gene (*ZmLox1*) were significantly induced in *zmerf061* mutant lines after infection with *E. turcicum*, indicating that *ZmERF061* is involved in modulating immune response through antagonizing the SA and JA/ET signaling pathways (Figure 7F). How *ZmERF061* modulates the JA and SA signaling pathways to improve plant resistance is an interesting question for future studies.

To ensure survival and negate the adverse effects of ROS, plants have evolved a complete antioxidant defense system to remove extra ROS (Radwan et al., 2010; Hu et al., 2017). POD and SOD are key antioxidant enzymes to help scavenge extra ROS in plants, so that ROS are maintained at a low level to improve the plant resistance against pathogens (Mengiste, 2012). Overexpression of *GmSnRK1.1* in soybean showed enhanced resistance to *P. sojae* through increasing the activity levels of SOD and POD, and *GmSnRK1.1*-RNAi plants exhibited opposite patterns (Wang et al., 2019). Our studies also showed that both SOD and POD activities were significantly lower in *zmerf601* mutant lines after infection with *E. turcicum* than those in the WT plants (Figures 7A,B), suggesting that *ZmERF061* may improve the resistance to pathogen in maize via regulating the plants’ antioxidant defense system.

Recently, some ERF genes have shown to be involved in plant defense reactions through interacting with other proteins (Meng et al., 2013; Dong et al., 2015). GmERF5 and GmERF113 interacted with GmbHLH to improve soybean resistance against *P. sojae* (Dong et al., 2015; Zhao et al., 2017). ORA59 could enhance *Arabidopsis* resistance against *Pectobacterium carotovorum* through interaction with RAP2.3 (Kim et al., 2018). In addition, several ERF genes can interact with MPK genes and are the substrates of pathogen-responsive MAPK signaling cascade (Popescu et al., 2009; Son et al., 2012; Cao et al., 2018). AtERF6 or AtERF104 could interact physically with AtMPK6 and be phosphorylated by AtMPK6 (Bethke et al., 2009; Meng et al., 2013; Wang et al., 2013). The upregulation of AtERF6 or AtERF104 in response to *B. cinerea* depends on AtMPK6 signaling cascade. Here, we found that ZmERF061 interacted physically with ZmMPK6-1 in yeast cells and *N. benthamiana* (Figure 8). We speculated that ZmERF061 may play an important role downstream of the ZmMPK6-1 signaling cascade in regulating maize defense against *E. turcicum*.

An increasing number of evidence indicated that ERF TFs can specifically bind to the GCC-box element to activate the expression of defense-related genes. AtERF1, ORA59, AtERF6, or AtERF96 could directly bind to the GCC-box element in the promoter of *AtPDF1.2* to enable its transcription activity (Berrocal-Lobo et al., 2002; Lorenzo et al., 2003; Pré et al., 2008; Moffat et al., 2012; Catinot et al., 2015). GmERF3, TiERF1, or VaERF057 also conferred the ability to bind to the GCC-box element (Liang et al., 2008; Zhang et al., 2009; Sun et al., 2016). In this study, we found that ZmERF061 specifically binds to the GCC-box element by Y_1_H Gold yeast strain and *in planta* (Figure 5). These results suggested that ZmERF061 may directly activate the expression of downstream defense-related genes by interacting with the GCC-box element in their promoter regions. It will be helpful to characterize the direct target genes that are regulated by ZmERF061 during immune response against *E. turcicum*.

In conclusion, we isolated and characterized a novel ERF gene, *ZmERF061*, which was a nucleus-localized transcription activator and could specifically bind to the GCC-box element. The expression of *ZmERF061* was induced by *E. turcicum*, SA, and MeJA. We also demonstrated that *ZmERF061* plays a positive role in modulating plant resistance to *E. turcicum* through regulating the expression of downstream defense-related genes and antioxidant defense system. Moreover, we found that ZmERF061 can interact with ZmMPK6-1. These data will be important to elucidate the function and regulatory mechanisms of *ZmERF061* in maize and provide a reference for breeding disease-resistant varieties.

## Data Availability Statement

The datasets presented in this study can be found in online repositories. The names of the repository/repositories and accession number(s) can be found in the article/ Supplementary Material.

## Author Contributions

LJ and WGY conceived and designed the experiments, contributed reagents, materials, and analysis tools. ZZ, ZW, and FZ performed the experiments and drafted the manuscript. WY, JC, and XR analyzed the data. All authors contributed to the article and approved the submitted version.

## Conflict of Interest

The authors declare that the research was conducted in the absence of any commercial or financial relationships that could be construed as a potential conflict of interest.

## Footnotes

1 http://www.ncbi.nlm.nih.gov/

2 https://www.expasy.org/

3 http://www.phytozome.net/maize; http://www.cbs.dtu.dk/serv-ices/signalp/
