# Supplementary material for: Maize Ethylene Response Factor ZmERF061 Is Required for Resistance to Exserohilum turcicum
Source: Front Plant Sci. 2021 Mar 9;12:630413. doi: 10.3389/fpls.2021.630413 (PMC7985547; doi:10.3389/fpls.2021.630413)
Supplement: Supplementary file 1 [file Table_1.docx]

**Table S1. The primer sequences used in this study.**

| Cloning of *ZmERF061* | *ZmERF061*-F | CCCATGGTGGACATGGCATTCACC |
| --- | --- | --- |
|  | *ZmERF061*-R | CCCACGTGAGTGACGGTGAGCTGGG |
| BiFC | *ZmERF061*-cYFP-F | CGCGGATCCATGGCATTCACCGGAGACGC |
|  | *ZmERF061*-cYFP-R | CCCTCGAGAGTGACGGTGAGCTGGGGAA |
|  | *ZmMPK6-1*-nYFP-F | CGCGGATCCATGGACGGCGGGGGGCAGCC |
|  | *ZmMPK6-1*-nYFP-R | CCCTCGAGCTGGTAATCTGGGTTGAATG |
| Yeast | pGADT7-*ZmERF061*-*F* | CGGAATTCATGGCATTCACCGGAGACGC |
| one-hybrid | pGADT7-*ZmERF061*-R | CGCGGATCCTCAAGTGACGGTGAGCTGGGGA |
|  | *ZmTub*-QF | CTACCTCACGGCATCTGCTATGT |
|  | *ZmTub*-QR | GTCACACACACTCGACTTCACG |
|  | *ZmERF061*-QF | GACATCACCTTCCCCGTTC |
|  | *ZmERF061*-QR | CTCGCCCATGTACTCGTTG |
|  | *ZmPR1a*-QF | GGCGAGAGCCCCTACTAGAC |
|  | *ZmPR1a*-QR | AAATCGCCTGCATGGTTTTA |
| qRT-PCR | *ZmPR10.1*-QF | AGATCACTAAAGCCAAGGAGTC |
|  | *ZmPR10.1*-QR | CATGGTCTAGTTGTAGGCTTCC |
|  | *ZmPR10.2*-QF | AGCCTTCAGCTAGCCCCAAGTT |
|  | *ZmPR10.2*-QR | GCGGAGGCCATTACTACTTCAG |
|  | *ZmLox1*-QF | ACGTGAACGACTACTGCTGG |
|  | *ZmLox1*-QR | TCATGGTGTCACGGTAGTGC |
| Subcellular | *ZmERF061*-GFP-F | GGGGTACCATGGCATTCACCGGAGACGC |
| Localization | *ZmERF061*-GFP-R | GCTCTAGAAGTGACGGTGAGCTGGGGAAAG |
|  | pGBKT7-*ZmERF061*-F | CGGAATTCATGGCATTCACCGGAGACGC |
| Yeast | pGBKT7-*ZmERF061*-R | CGCGGATCCTCAAGTGACGGTGAGCTGGGGA |
| two-hybrid | pGADT7-*ZmMPK6-1*-F | CGCGGATCCATGGACGGCGGGGGGCAGCC |
|  | pGADT7-*ZmMPK6-1*-R | CCCTCGAGCTGGTAATCTGGGTTGAATG |

| LUC Activity | pGreenII-62-ZmERF061-F | CGCGGATCCATGGCATTCACCGGAGACGC |
| --- | --- | --- |
| Assay | pGreenII-62-ZmERF061-R | CGGAATTCTCAAGTGACGGTGAGCTGGGGA |
